# ASO Author Reflections: Residual Breast Tissue After Mastectomy: Prevalence, Patterns, and Predictors

**DOI:** 10.1245/s10434-025-17534-z

**Published:** 2025-06-03

**Authors:** Marcin Jakub Napierała, Wojciech Olszewski, Magdalena Rosińska, Magdalena Okarska-Napierała, Paweł Winter, Aleksander Grous, Zbigniew Nowecki

**Affiliations:** 1 Department of Breast Cancer and Reconstructive Surgery, Maria Skłodowska-Curie National Research Institute of Oncology, Warsaw, Poland; 2 Department of Pathology and Laboratory Diagnostics, Maria Skłodowska-Curie National Research Institute of Oncology, Warsaw, Poland; 3 Department of Computational Oncology, Maria Skłodowska-Curie National Research Institute of Oncology, Warsaw, Poland; 4 Department of Pediatrics with Clinical Assessment Unit, Medical University of Warsaw, Warsaw, Poland; 5 Surgical Oncology Department, Research and Development Centre, Regional Specialist Hospital in Wroclaw, Wrocław, Poland

## Past

Residual breast tissue (RBT) remaining after mastectomy is a potential concern due to its unclear prevalence and clinical implications. While previous studies have attempted to characterize RBT, they have been limited by small sample sizes, inconsistent sampling methods, and a narrow focus on selected mastectomy types. Furthermore, the factors that may influence the presence of RBT—such as surgical technique, breast size, or surgeon experience—have remained poorly studied.^1–4^ Our study aimed to comprehensively evaluate the incidence of RBT across all four common types of mastectomy [modified radical mastectomy (MRM), simple mastectomy (SM), skin-sparing mastectomy (SSM), and nipple-sparing mastectomy (NSM)] using a standardized histopathological sampling protocol.

## Present

In our prospective analysis of more than 400 mastectomies, we found that RBT was present in approximately one-third of all procedures, regardless of mastectomy type. Notably, in nipple-sparing mastectomy (NSM), RBT was almost universally detected beneath the nipple-areolar complex (NAC), consistent with previous findings. Importantly, our results indicate that the prevalence of RBT within the remaining skin flap is comparable across mastectomy types. We also identified key risk factors for RBT, including smaller breast volume and a history of prior breast surgery. Surgeon experience—particularly in NSM—also appeared to influence outcomes, underscoring the importance of surgical expertise in achieving optimal oncological safety.^5^

## Future

While our findings help establish the baseline prevalence and predictors of RBT, the long-term oncological significance of residual tissue remains uncertain. Future research should focus on correlating RBT volume with local recurrence and survival outcomes, particularly in high-risk populations. Standardizing histopathological sampling could also enhance clinical decision-making. Ultimately, integrating RBT evaluation into post-mastectomy surveillance protocols—especially in risk-reducing settings—may guide tailored patient follow-up and improve overall outcomes.
